# Analysis of Advanced Driver-Assistance Systems for Safe and Comfortable Driving of Motor Vehicles

**DOI:** 10.3390/s24196223

**Published:** 2024-09-26

**Authors:** Tomasz Neumann

**Affiliations:** Faculty of Navigation, Gdynia Maritime University, 81-225 Gdynia, Poland; t.neumann@wn.umg.edu.pl

**Keywords:** transportation, advanced driver-assistance systems, autonomous transport, safety of transport

## Abstract

This paper aims to thoroughly examine and compare advanced driver-assistance systems (ADASs) in the context of their impact on safety and driving comfort. It also sought to determine the level of acceptance and trust drivers have in these systems. The first chapter of this document describes the sensory detectors used in ADASs, including radars, cameras, LiDAR, and ultrasonics. The subsequent chapter presents the most popular driver assistance systems, including adaptive cruise control (ACC), blind spot detection (BSD), lane keeping systems (LDW/LKS), intelligent headlamp control (IHC), and emergency brake assist (EBA). A key element of this work is the evaluation of the effectiveness of these systems in terms of safety and driving comfort, employing a survey conducted among drivers. Data analysis illustrates how these systems are perceived and identified areas requiring improvements. Overall, the paper shows drivers’ positive reception of ADASs, with most respondents confirming that these technologies increase their sense of safety and driving comfort. These systems prove to be particularly helpful in avoiding accidents and hazardous situations. However, there is a need for their further development, especially in terms of increasing their precision, reducing false alarms, and improving the user interface. ADASs significantly contribute to enhancing safety and driving comfort. Yet, they are still in development and require continuous optimization and driver education to fully harness their potential. Technological advancements are expected to make these systems even more effective and user-friendly.

## 1. Introduction

The number of cars equipped with modern systems that highly support drivers increases yearly. Many science fiction authors wrote about it, and this motif also appeared in films of this genre—about cars moving without a driver. This fanciful vision is now becoming a reality. All this is due to the dynamic development of the automotive industry, particularly ADASs (advanced driver-assistance systems), which turn our cars into vehicles that move without the participation of a driver.

ADAS—a set of advanced driver-assistance systems. No single solution can be called ADAS, and it is more of an idea than a tool. In line with this idea, various sets of driver assistance systems are created, the purpose of which is to support the person driving the vehicle by:

- increasing driving safety;
- improvement of driving comfort.

Currently, we have many ADASs, each having different complexity, values, and components [1]. There are really simple ADASs, based solely on the operation of a video recorder (camera), and almost space-like ones, where components of the drive, suspension, and braking systems cooperate with each other, as well as a whole network of complex sensors. Therefore, this paper analyzes in detail and describes only some ADASs [2]—the most important, as well as those that have the greatest impact on improving driver safety [3]. This paper will refer to practical and theoretical aspects because advanced driver-assistance systems are very complex systems, and their intricate design is constantly enriched with innovative solutions. The materials relied primarily on professional literature, scientific publications, articles from industry magazines, and materials publicly available on websites. As part of this paper, a survey was conducted to collect drivers’ opinions on their experiences using these technologies and how they affect driving safety and comfort. The third chapter describes the detailed survey methodology and presents the results and conclusions.

The ADAS will undoubtedly control the future development of the automotive industry. This is indicated, among others, by orders of state and EU administrations. Changes to EU law were proposed by the European Commission and supported by the European Parliament and representatives of Member States, which assume that from 2022, every car of a new model or generation should be equipped with the ADAS. For example, passenger cars will have to be equipped with a speed limit assistant, a lane-keeping system, and a tire pressure measurement system, while trucks and buses will have an advanced emergency braking system. Moreover, starting in 2024, all cars must be equipped with the so-called “black boxes” recording events and trucks—with the new BLIS (blind spot information system), improving cyclists’ safety in the so-called blind spot. Two years later, the requirement to use driver fatigue detection systems will also come into force. At the latest, starting in 2029, the requirement to introduce the DVS (direct visibility standard) in trucks will come into force, which requires the use of cameras or additional mirrors [4]. Although the abovementioned regulations have not yet been officially adopted and are subject to further legislative procedures, we can confidently expect their approval [5].

The standard ADAS, with which modern cars are increasingly equipped at the factory, includes active cruise control, lane keeping assistant, blind spot monitoring system, road sign recognition system, and adaptive headlight system [6]. Increasingly, the set of ADASs also includes a traffic jam assistant or a parking assistance system.

## 2. Description and Operation of Sensors in Advanced Driver-Assistance Systems

**Figure 1:**
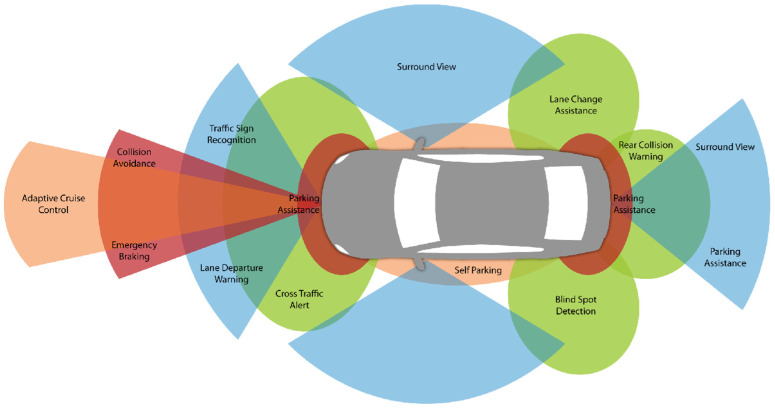
Location of ADASs in the vehicle [11].

Advanced driver-assistance systems operate primarily by observing the environment in which the car is moving [7]. This requires numerous sensors and cameras. Efficiently functioning sensors improve safety, increase situational awareness, and reduce the risks associated with driving. These systems alert the driver when potential road hazards are detected [8] (see Figure 1).

The data are analyzed and processed by software that uses advanced algorithms to identify potential threats or situations requiring the driver’s attention. Some systems require information from several sensors simultaneously to function properly. Based on these analyses, ADASs can generate warning signals, activate driving assistant or emergency braking, and even take autonomous actions to prevent collisions or minimize the consequences of accidents [9]. The ADAS driver-assistance system package may have different sensor configurations depending on the car’s age, equipment level, selected version, and whether it belongs to a specific segment [10].

### 2.1. Radar Sensors

Radars are very important elements of the ADAS. As the definition suggests, this device works thanks to the ability to receive radio waves. Initially, radars were important primarily in military operations—for detecting planes, ships, and missiles, determining the direction of movement of objects, and assessing distance, size, and measuring speed. Over time, the advantages of these devices have also been noted in more peaceful fields, such as air or surface traffic control, and they are also useful in meteorology (for detecting clouds), geology (for penetrating the ground), glaciology (studying glaciers), geophysics, or astronomy [12]. We cannot forget radars’ importance in road safety in all countries—they are a basic tool for police work, regardless of geographical location [13].

Radar sensors are devices that use electromagnetic waves to detect objects in space. For this purpose, the radar sends a beam of microwave signal, which is then reflected from objects. The echo, or reflected signal, returns to the radar, which is later filtered and processed. Although the operating principle of all radars is based on similar assumptions, the data collected by individual radars differ significantly. Sensors placed in cars can precisely determine the vehicle’s surroundings. Radar reflection analysis provides information about the type of object from which the electromagnetic wave was reflected. The waves are reflected non-uniformly, depending on the material the object is made of, its shape and, above all, its predisposition to reflect electromagnetic waves. Based on a detailed analysis of reflection data, engineers create precise detection algorithms that help determine the type of object, e.g., pedestrians, cyclists, sidewalk curbs, or road signs [14]. This and similar information are collected by the radar in real time and then delivered to subsequent components of the car’s onboard computer. The provided information is then processed and transported to other modules that improve the safety and comfort of car users. Radar sensors are installed in passenger cars near the front license plate. This location of the radar allows for precise observation of the car’s surroundings [15]. To increase the precision of the radar, it is often combined with other sensors, e.g., cameras, which allow for a more accurate analysis of the space in which the car is moving [16].

### 2.2. A Camera or Set of Cameras

Cameras are usually placed on the windshield. This is the most popular and cheapest solution, on which the simplest ADASs are also based. In more advanced systems, it is only an important element, but not the only one. In more complex ADASs, we deal with many cameras that provide a full 360° overview of the field around the vehicle. Cameras of this type are placed in the front bumper, grill, under the side mirrors, or the trunk lid. The possibility for drivers to purchase and install cameras on their own on the windshield has contributed to the growing popularity of cameras and, consequently, to improved road safety. These devices have very good optical sensors and image processors, which can be used effectively. The cameras can analyze hundreds of details on the route that the driver may miss while driving.

Cameras used in ADASs often operate on the principle of digital image processing, which enables precise data collection and processing. A standard camera system consists of optical components, including a lens that collects light rays and transforms them into an image on a photosensitive matrix. The size of the matrix and the type of sensor used, i.e., CMOS or CCD, are important in converting light into an electrical signal. The information from the matrix is then processed by an electronic system that digitally converts the image and transmits it as data to the ADAS.

Cameras in ADASs play an essential role in improving road safety by offering many functions. The first is the recognition of road signs, including speed limit signs, stop signs, and turn restrictions. The ADAS provides the driver with up-to-date information about road regulations, preventing potential violations. Another function of the cameras is lane monitoring, which allows the driver to be warned of dangers, such as approaching a double solid line. The cameras also detect other vehicles and pedestrians, allowing the system to respond to potential threats, such as activating emergency braking or displaying warnings. The cameras are also useful when parking, providing images of the front and rear of the vehicle on a screen in the cabin. This facilitates precise maneuvering when parking, preventing collisions. Additionally, cameras can monitor driver behavior, detecting fatigue and suggesting the need for a break. The last important function is maintaining the appropriate distance and adaptive cruise control, which uses cameras to control the distance from the vehicle in front and adapt the speed to the traffic on the road. This ensures a smoother and safer ride, especially in difficult traffic conditions.

### 2.3. LiDAR (Light Detection and Ranging)

It is a combination of a laser and a telescope; the device works on a similar principle to radar but uses a laser light beam instead of radio microwaves [17]. The principle of its operation is based on sending light pulses of a specific wavelength. The light scattered along the way is constantly observed using a telescope and recorded using a detector, photomultiplier, and cameras. Finally, the data go to the computer module, which is analyzed in detail. The principle of operation of LiDAR technology allows for much greater precision compared to radar technologies currently installed in new cars and designed to analyze the vehicle’s surroundings [18]. Currently, two technologies are mainly used. One involves using a short laser pulse—up to 150,000 pulses can be generated per second. If such an impulse hits an object, e.g., another car, it is reflected, and the distance from the object, its position, and ultimately its shape are determined based on the so-called time of flight. The second technology involves emitting light continuously. The phase change between the received and sent pulse is analyzed in this case. Radio waves have much less absorption than light waves when in contact with various objects. Thanks to this, LiDAR technology can obtain a much higher resolution and thus “draw” a much more accurate terrain map with more details [19]. High-class LiDAR sensors can recognize details of a few centimeters at a distance of more than 100 m. LiDAR sensors also have disadvantages, i.e., a very specific range of “visibility” with a given resolution. A single sensor can collect data from the car’s surroundings with an accuracy of a specific distance from the vehicle, e.g., up to 30 m. The second sensor will have “visibility” in the 30–200 m range. In complex systems, one LiDAR module may contain up to several sensors, so that it can exclude the mentioned defect and be able to provide observations of the entire desired space around the car [20].

### 2.4. Ultrasounds

Ultrasound is sound waves whose frequency is too high for humans to hear. The frequency of 20 kHz is considered to be the upper limit of audible frequencies and the lower limit of ultrasound, although, for many people, this limit is much lower. The conventional upper limit of ultrasound is the frequency of 1 GHz. Ultrasonic sensors in ADASs emit ultrasonic waves, which are then received by the sensor after reflection from an obstacle. By analyzing the return time of these waves, the system can precisely determine the distance to detected objects. Ultrasonic waves with a frequency above 20 kHz allow for accurate measurements at close range, which is crucial for many applications in driver assistance systems [21]. These sensors have a limited measurement range, typically effective from a few centimeters to several meters. This limitation is offset by strategically placing multiple sensors around the vehicle, allowing for more complete coverage of the space around it. In practice, ultrasonic sensors are used in various aspects of driver assistance, including parking assistance systems, detection of obstacles in the blind spot, and assistance in low-speed maneuvers. 

Their integration with other sensor systems, such as cameras, radars, or LiDARs, creates more comprehensive and effective ADASs. This synergy of technologies provides a more comprehensive picture of the road situation, which is essential for the precise operation of driver assistance systems. Despite many advantages, ultrasonic sensors also have their limitations. Their effectiveness may be limited in fast and dynamic driving scenarios due to their relatively short operating range. Additionally, different weather conditions, such as rain or snow, can affect the effectiveness of these sensors. Another challenge is acoustic interference in urban environments due to other sound sources. Ultrasonic sensors are an important element of modern ADASs, offering precise information about the vehicle’s surroundings, although external and technological factors may limit their effectiveness. The development of ultrasonic technologies and their integration with other sensor systems will continue to be key to further developing and improving ADASs.

## 3. The Most Popular Advanced Driver-Assistance Systems Found in Cars on European Roads

Modern vehicles are equipped with several advanced driver-assistance systems that aim to improve travel safety, comfort, and efficiency. As technology develops at an accelerating pace, these systems are becoming not only more advanced but also more common in new vehicles on European roads.

### 3.1. Adaptive Cruise Control (ACC)

**Figure 2:**
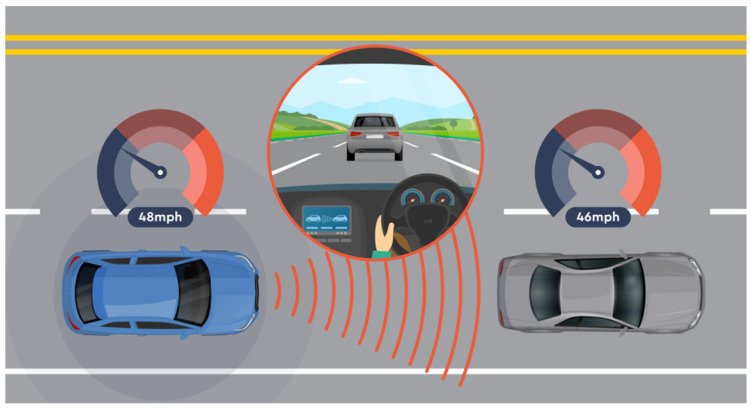
Vehicle speed adjustment system in cruise control (ACC) [22].

The vehicle’s speed control system, commonly called cruise control, allows you to automatically maintain a constant speed without manually using the accelerator pedal (Figure 2). Popular in modern vehicles, it offers great convenience, especially when travelling in areas with speed limits, such as motorways, where it is necessary to maintain a steady driving pace for long periods. ACC uses advanced sensors such as radar and cameras to monitor the distance to other vehicles and adjust speed to maintain a safe distance.

Engineer Ralph Teetor made the first steps towards the invention of cruise control in the 1950s. The inspiration for Teetor was the desire to ensure a smooth ride, which was a response to the observation of a driver who frequently changed the speed of his vehicle. In 1958, Chrysler introduced the first commercially available speed control system, called “Auto-Pilot”. Cruise control works by electronically controlling the vehicle components responsible for acceleration, most often via the engine [23]. When the driver activates the cruise control and sets the desired speed, the system automatically adjusts the engine power and, in some cases, the transmission to maintain a constant vehicle speed. This happens regardless of changes in the road topography, such as inclines or declines. Cruise control operation is based on using various sensors that monitor the vehicle’s speed and maintain it at a set level [24]. Modern versions of cruise control often offer more advanced features, such as adaptive cruise control (ACC). ACC automatically adjusts the vehicle’s speed to the speed of the vehicle in front, using radar sensors or cameras to monitor the distance to other vehicles. In this way, the system can adjust the speed accordingly, maintaining a safe distance from vehicles in front of it. When the Active Cruise Control (ACC) system is activated, the driver can set his preferred maximum speed and distance from the vehicle in front of him. The ACC system automatically takes control of the vehicle, managing speed, acceleration, deceleration, and braking depending on road conditions. A key component of ACC is the radar measurement unit, designed to monitor nearby vehicles’ distance and speed. Despite its advanced functions, the ACC system is not fully autonomous. It works with other vehicle systems, using a variety of signals to ensure that the set speed and distance are maintained safely and efficiently [25]. However, the most common cruise control in cars today is the conventional cruise control, also known as a static speed control system, which allows the driver to set and maintain a constant vehicle speed without manually controlling the accelerator pedal. This system does not respond to changes in road traffic conditions and requires manual intervention by the driver if it is necessary to change speed. An evolution in cruise control is the cruise control with the Stop and Go function, which allows the vehicle to automatically stop and start again in changing traffic conditions, which is particularly useful in city traffic jams. This system takes control of braking and acceleration in congested traffic, significantly reducing the driver’s workload when driving in congested conditions. It also helps to increase fuel efficiency by minimizing unnecessary acceleration and braking. Another new feature in cruise control is predictive cruise control, which uses data from the GPS navigation system and vehicle sensors [26]. This cruise control adjusts your speed based on upcoming changes in the road topography, such as curves, inclines, or declines. This system contributes to optimizing fuel consumption and increasing safety [25]. 

Cruise control and its evolutions indicate a growing trend towards greater vehicle automation. They make driving safer, more comfortable, and more energy-efficient. It is worth emphasizing that although ACC systems are advanced, they still require vigilance and intervention from the driver, as they are not fully autonomous systems. Their main task is to support the driver, not to take complete control of the vehicle.

### 3.2. Blind Spot Detection System—BSD

**Figure 3:**
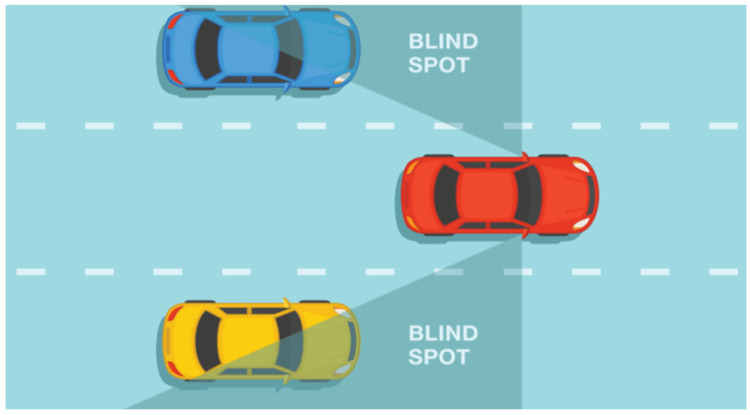
Blind spot detection (BSD) [27].

Another advanced driver-assistance system is blind spot detection systems (BSD), an important part of innovations to increase road safety (Figure 3). These systems are designed to minimize the risk of vehicles going unnoticed by the driver in hard-to-see areas around the vehicle, known as blind spots. BSD is a solution aimed at increasing safety when changing lanes. This system monitors areas invisible to the driver and warns him of vehicles in the blind spot.

The blind spot detection system (BSD) is a complex electronic system whose structure includes sensors, a signal processor, and a user interface. The central component of the blind spot detection system is sensors, usually located in key vehicle locations, such as the rear bumpers. These sensors, which operate on the radar or ultrasonic principle, play a fundamental role in the system. They emit radio waves in the case of radars and ultrasonic waves in the case of ultrasonic sensors, which propagate around the vehicle. When these waves encounter objects in the vehicle’s blind spot, they are reflected from them and return to the sensors. The feedback obtained from wave reflection provides data about objects in the blind spot, such as their presence, position, and possible movements. The collected signals are then transmitted to the vehicle’s central processor, the brain of the entire system. This processor, equipped with advanced computational algorithms, analyzes this data in a detailed and complex way. During this processing process, the algorithms focus on identifying objects that are in the vehicle’s blind spot and are not visible to the driver. However, it is not limited to detecting these objects’ presence. The processor also analyzes additional parameters, such as the object’s distance from the vehicle, speed, and movement trajectory. This multidimensional analysis allows for a thorough understanding of the situation in the blind spot, which is crucial for assessing whether a given object poses an actual collision risk. By assessing all these factors, the system can provide the driver with precise and up-to-date information necessary to make quick and safe driving decisions.

The blind spot detection system communicates with the driver through an integrated, multi-dimensional user interface that provides intuitive and immediate notifications [28]. The most common forms of communication include visual signals, such as specially designed indicators on side mirrors that can illuminate or flash to alert the driver to the presence of a vehicle in a blind spot [29]. Alternatively, the system can use audible signals, such as a warning tone that is activated when the driver signals to change lanes and another vehicle is in the blind spot. Additionally, some systems use haptic signals, such as steering wheel vibration, which can be particularly effective because they provide direct physical feedback that the driver can feel immediately. As part of the development of blind spot detection (BSD) systems, we encounter several technological and operational challenges that are crucial to their effectiveness and reliability. The first is the adaptation of these systems to various environmental conditions. BSD systems must maintain effectiveness in various scenarios, including severe weather conditions such as heavy rainfall, snow, fog, and strong sunlight. To meet this challenge, it is necessary to develop sensors and algorithms resistant to changing atmospheric and optical conditions. The second challenge is to minimize the number of false alarms, as too frequent false alarms can lead drivers to ignore warnings, which poses a safety risk. The third challenge is integrating BSD systems with various vehicles, from cars to trucks. This diversity requires adapting the technology to the specifications of different vehicles, including blind spot size and installation requirements. Another issue is ensuring the compatibility of BSD systems and the effective cooperation of other vehicle security systems. This requires coordination and cooperation between hardware and software manufacturers to achieve consistency and efficiency across the vehicle security system. 

The blind spot detection system is an important element of modern driver assistance technologies, significantly contributing to increased road safety. Its effectiveness depends on the precise integration of advanced technological components and the effective solution of technical and operational challenges. Despite the challenges to be overcome, BSD systems represent an important step towards more advanced and safer vehicles on the road.

### 3.3. Lane Maintenance System—LDW/LKS

**Figure 4:**
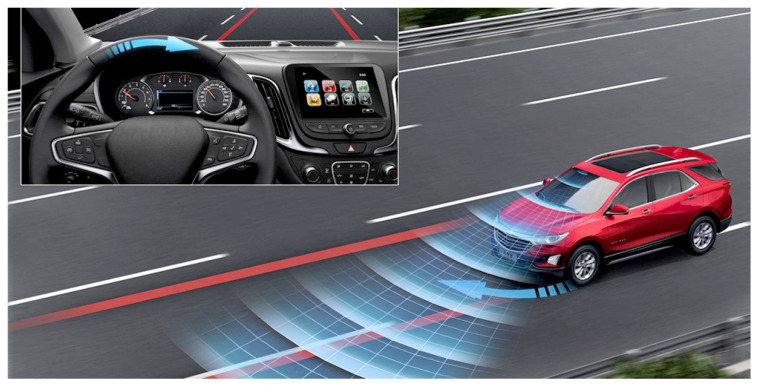
Belt maintenance system—LDW/LKS [27].

The LDW (lane departure warning) and LKS (lane keeping system) systems are important elements of driver assistance (Figure 4). They aim to increase road safety by helping to keep the vehicle in the appropriate lane. LDW warns the driver of unintentional departure from the lane while LKS actively corrects the path to prevent the driver from leaving. 

LDW is an advanced driver assistance technology. The main function of this system is to prevent the vehicle from accidentally leaving the lane. It monitors the vehicle’s position relative to the lane markings using various sensors and cameras [30]. LDW, as an element of the system, is intended to warn the driver about unintentional lane changes through visual, acoustic or vibration signals. In turn, LKS actively engages in the driving process, correcting the driving path to keep the vehicle in the right lane. These systems, initially available only in high-end car models, have, over time, become standard equipment in an increasing number of vehicles. Progress in sensor and computer technologies played an important role in developing and increasing these systems’ effectiveness, allowing for their wider adaptation and improvement. 

The LDW and LKS systems represent a significant advancement in automotive technology based on advanced image processing algorithms. These algorithms, which constitute the system’s core, analyze data from cameras placed on vehicles. These cameras are designed to precisely monitor the lines on the road, allowing the vehicle’s position about these lines to be accurately determined. The functioning of the LDW system is based on continuous tracking and analysis of the road line. A warning mechanism is activated when the system detects that the vehicle is unintentionally approaching or crossing the lane boundary. This mechanism can take many forms—from audible signals through visual warnings on the dashboard to vibrations in the steering wheel. The purpose of these signals is to draw the driver’s attention to potential danger and encourage him to correct his driving path. The LKS system, in turn, is a step further towards automation and safety. In addition to warning the driver, LKS actively engages in the driving process. If it detects that you are approaching the lane boundary, the system automatically corrects the steering wheel. This action is intended to gently but effectively guide the vehicle back to the center of the lane, minimizing the risk of collision. This intervention is subtle so that the driver can take control of the vehicle at any time, which is a key aspect of safety. In addition to monitoring the road line, modern LDW/LKS systems often integrate additional functions, such as road sign recognition or driver behavior analysis, further increasing their effectiveness. These systems have evolved from simple warning solutions to more advanced ones that offer assistance in controlling the vehicle. We distinguish:

- Passive LDW systems that only warn of danger;
- Active LDW systems that combine warnings with light steering or braking corrections;
- LKS systems with full control that signal danger and actively keep the vehicle in the lane.

The effectiveness of LDW/LKS systems largely depends on their ability to adapt to changing weather conditions, such as rain or snow. Modern versions of these systems use advanced algorithms that adapt camera operation and image processing to reduced visibility. As a result, even when road lines are partially obscured, these systems remain effective, ensuring continuous safety. So, LDW and LKS systems are key technologies in road safety, assisting the driver in keeping the vehicle in its lane and increasing overall road awareness.

### 3.4. Intelligent Headlamp Control—IHC

**Figure 5:**
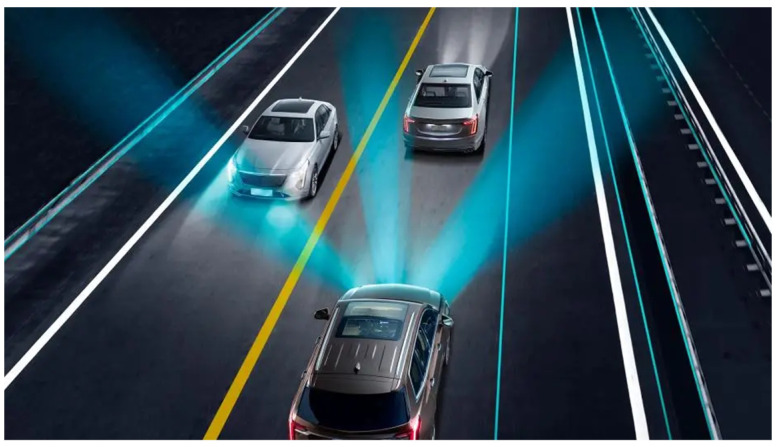
Intelligent headlamp control (IHC) [31].

Intelligent lamp control system, better known as intelligent headlamp control (IHC), is a system that automatically adjusts the vehicle’s lighting to road conditions (Figure 5). Thanks to sensors and cameras, this system regulates the light intensity and direction, improving the visibility of the driver and other road users [20].

Increasingly used in modern vehicles, IHC is taking an important step towards raising safety standards and driving efficiency. The main function of this system is the automatic adaptation of the vehicle’s external lighting, which includes adjustment of light intensity and direction. A key aspect of IHC operation is its ability to dynamically respond to changing environmental and road traffic conditions, which allows for optimization of visibility and minimization of risk to the safety of the driver and other road users. In response to varied and often unpredictable road conditions, this system automatically adjusts the lighting, which is especially important when driving at night, in bad weather conditions, or in changing traffic intensity. The origins of the intelligent headlamp control (IHC) date back to the beginning of the 21st century, when the need to improve safety during night driving increased. At that time, IHC, in its original form, was limited to automatic switching between low and high beams. Over time, thanks to progress in sensor technologies and the development of visual processing algorithms, the capabilities and scope of operation of the IHC system have expanded significantly. The operating mechanism of the intelligent headlamp control (IHC) is based on advanced interaction between various technological components of the vehicle. The integrated sensor system plays the main role here, including cameras, light, and motion sensors. These sensors are constantly active, scanning the vehicle’s surroundings to identify any significant changes in road and environmental conditions. The information collected by the sensors is transferred to the vehicle’s central computer system, which uses complex algorithms to analyze and interpret the data. Based on this analysis, the computer system makes decisions regarding appropriate lighting regulation, including light intensity and direction. For example, if an oncoming vehicle is detected, IHC can automatically switch the headlights from a high beam to a low beam to avoid dazzling the oncoming driver. An important aspect of IHC operation is its ability to adapt to various weather and lighting conditions. In situations where weather conditions such as fog or heavy rain limit visibility, IHC can reduce light intensity, which helps improve visibility while minimizing the risk of blinding other road users. Additionally, some advanced versions of IHC can adjust the direction of light depending on the vehicle’s direction, steering angle, and even driving speed. This means that when turning or maneuvering around corners, the system can actively direct light in the direction the vehicle is moving, providing better visibility.

There are three types of IHC systems:

- Systems with automatic headlight switching—the simplest form of IHC involves automatic switching between low and high beams. The main purpose of this type of system is to prevent dazzling oncoming drivers by automatically reducing light intensity in appropriate situations;
- Adaptive front lighting systems—these are advanced IHC systems that adjust the angle and intensity of the headlights depending on various factors, such as vehicle speed, driving direction, and general environmental conditions. They are designed to provide optimal road illumination, increasing driving safety;
- Cornering lighting systems—these systems illuminate the road when maneuvering around bends. These systems actively direct light in the direction the vehicle is facing by directing the headlights according to the steering angle. They provide better visibility around corners and at intersections, which is especially useful when driving at night.

Intelligent lamp control systems represent a significant advancement in automotive technology, increasing road safety through better adaptation of lighting to changing road conditions. Its ability to dynamically adapt to a variety of driving scenarios makes it a key component of modern vehicles, raising standards of driving safety and efficiency.

### 3.5. EBA Driving Assistance System

**Figure 6:**
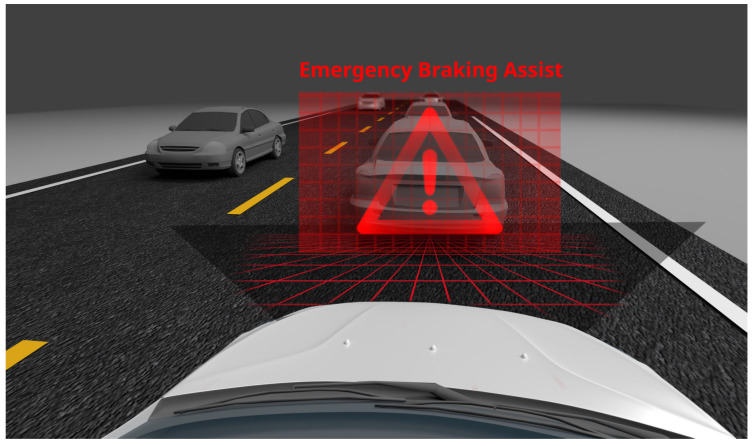
Emergency brake assist [32].

EBA (emergency brake assist) is a system that reacts automatically in sudden braking situations (Figure 6). If a collision threat is detected, this system helps the driver apply appropriate braking force, which can significantly shorten the braking distance and avoid an accident.

In today’s rapidly evolving automotive world, where roads are increasingly congested and the risk of road accidents continues to increase, there is an urgent need to develop effective safety technologies. In this context, the idea of creating the emergency brake assist (EBA) system was born, which is a response to the growing challenges related to road safety. EBA has been designed to support drivers in critical situations, minimizing the risk of collisions and their potentially tragic consequences. The origins of EBA date back to the 1990s, when engineers and car designers began to look for ways to use modern technologies to improve vehicle safety. The EBA system, combining advanced data processing algorithms and sensor technologies, was one of the first steps towards intelligent driver assistance. Developments in the field of electronics and computer data processing have enabled the creation of systems that can analyze the vehicle’s surroundings in real time and take immediate actions to prevent accidents. Its functioning is based on continuous monitoring of the vehicle’s surroundings, which is possible thanks to the use of a complex set of sensors. These sensors, which include radar, cameras, and ultrasonic sensors, scan the space around the car, providing key information about road conditions and the behavior of other road users. Based on the data collected by the sensors, the EBA system assesses the risk of potential collisions. It uses advanced algorithms that analyze vehicle speed, distance from objects, and road traffic patterns. When an increased risk situation is detected, EBA activates a series of actions aimed at minimizing this risk [33]. First, the system informs the driver about the threat through visual, acoustic, or vibration signals. This is a key element of EBA’s operation because it primarily focuses on the reaction of the person behind the wheel. However, if the driver does not react in time, the system enters the next phase of its operation, which includes braking support. In this phase, EBA increases the braking force that the driver has applied, with the aim of increasing braking efficiency and avoiding a collision. 

In critical situations when the driver is unable to react, some versions of the EBA system can even initiate the emergency braking process on their own. This aspect of the system demonstrates its ability to adapt to various driving scenarios and provides an additional layer of safety. A priority aspect of EBA is also its integration with other vehicle systems, such as collision warning systems and traction control. This collaboration increases the overall effectiveness of the system and allows for more comprehensive protection.

The EBA steering assistance system can come in various forms and levels of advancement, depending on the vehicle manufacturer and the specific car model. Although all versions of EBA have the same basic goal—collision prevention through emergency braking assistance—there are differences in the way each system accomplishes this. Here are some types of EBA steering assistance:

- Basic EBA—this is a type of EBA system that focuses mainly on increasing the braking force when it detects a sudden need for the driver to brake. It does not include advanced predictive features or is integrated with other vehicle safety systems;
- EBA with Threat Detection EBA—more advanced EBA systems not only support braking, but also actively monitor the vehicle’s surroundings to detect potential threats early. They can use sensors, radars, or cameras to assess the road situation and respond to threats early;
- Adaptive EBA—these systems are capable of adapting their response depending on driving conditions, such as vehicle speed, road conditions, and even the behavior of other vehicles on the road. They are more flexible and can provide a more varied response in different situations;
- Integrated EBA—these are systems that are integrated with other vehicle safety systems, such as collision warning systems, lane keeping systems, or adaptive cruise control. These integrated systems can offer a more comprehensive security solution by analyzing and responding to a wider range of sensory data;
- EBA with Autonomous Emergency Braking EBA—this is the most advanced form of EBA, which can initiate emergency braking on its own, even if the driver does not respond to warnings. This is particularly useful in situations where the driver’s reaction time may be insufficient to avoid a collision;

The assistance system is a technology that significantly contributes to increasing road safety. By automating the braking process, it not only supports the driver in critical situations, but can also prevent accidents that could have tragic consequences. Its integration with other safety systems and the continuous development of this technology significantly improve road safety.

To sum up, the above-mentioned ADASs significantly contribute to increasing driving safety and comfort. Their growing presence in modern vehicles indicates a trend towards greater automation and driver support, leading to safer and more comfortable journeys. At the same time, it is extremely important to consciously use these systems to maximize their potential while maintaining vigilance and responsibility for the driving process.

## 4. Assessment of the Effectiveness of ADASs in the Area of Safety and Driving Comfort in the Opinion of Drivers

In recent years, advanced driver-assistance systems have become an important element of modern vehicles, aiming to increase driving safety and comfort.

### 4.1. The Role of Driver-Assistance Systems in Improving Road Safety

ADASs are breakthrough solutions in the automotive field, opening new perspectives for improving road safety. Although road accidents are not directly related to the safety systems installed in vehicles, these systems can contribute to solving them. These solutions often save lives and contribute to reducing the number of accidents. Despite this, humans remain the main factor influencing road safety and are the key cause of road accidents [34]. In 2022, the number of accidents caused by the driver’s fault is 19,373, which exceeds 90% of all accidents in Poland. The cause of approximately 70% of them is the driver’s inappropriate reaction or lack thereof. ADASs installed in passenger cars make it easier to perform basic activities and contribute to increasing safety. Firstly, these systems provide drivers with valuable information about the surroundings and potential threats, and also intervene automatically in critical situations. An example is ACC cruise control, which automatically adapts the vehicle’s speed to the speed of the vehicle in front, maintaining a safe distance. This is an evolution of the classic cruise control, adapting its functionality to changing road traffic conditions. ACC can significantly reduce the risk of rear collisions by eliminating human errors associated with assessing distance and speed [35]. The blind spot detection (BSD) system informs the driver about vehicles in the blind spot, which is particularly useful when changing lanes. By reducing “invisible” areas, BSD helps reduce the risk of side collisions. Next, the lane keeping system (LDW/LKS) is designed to prevent unintentional departure from the lane. LDW warns the driver when the vehicle unexpectedly starts to cross the lane line, while LKS actively intervenes, correcting the vehicle’s driving path. These systems are particularly useful in preventing accidents caused by driver fatigue or inattention [11]. And the intelligent lamp control system (IHC) adjusts the vehicle’s lights to ambient conditions, improving visibility and safety at night. This system automatically switches between high beam and low beam, ensuring optimal visibility without dazzling other road users. Better night visibility significantly reduces the risk of accidents. Another is the steering assistance system (EBA), which, when a potential collision is detected, initiates automatic braking in order to avoid or mitigate the effects of the collision [36].

Despite its numerous benefits, ADAS also comes with some challenges and limitations. There is a risk of over-reliance on ADASs, which may lead to drivers neglecting their own responsibilities and vigilance. Additionally, these systems may sometimes generate false alarms or may not respond appropriately to all situations, which can create confusion and uncertainty. It is also important to ensure that ADASs operate effectively in a variety of road and weather conditions [37]. Analyzing the presented driver assistance systems, it can be seen that each of them makes a significant contribution to improving road safety. It is worth emphasizing, however, that these systems are only support for the driver and cannot fully replace his attention and skills. It is also important that the development of these technologies goes hand in hand with user education to ensure their conscious and effective use. Further development of these technologies is expected in the future, which may further increase the level of road safety. 

### 4.2. The Impact of Advanced Driver-Assistance Systems in Improving Driving Comfort

Advanced driver-assistance systems significantly contribute to improving driving comfort, offering a wide range of functions that make every day driving more efficient and easier. These systems reduce the physical and mental burden on car users. The introduction of ADAS is a step towards more intuitive and less stressful driving, which makes traveling more pleasant and safe for all road users. By using the latest technological achievements, ADAS minimizes the need for constant driver intervention. For example, ACC cruise control significantly increases driving comfort, especially on long journeys and in congested traffic. Automatic speed adjustment and maintaining a safe distance from the vehicle in front reduces the need for constant driver intervention, minimizing fatigue and stress. Meanwhile, the blind spot detection (BSD) system increases driving comfort by increasing the driver’s confidence during maneuvers such as changing lanes or overtaking. With blind spot warnings, drivers can make maneuvers with greater confidence, reducing the stress associated with the possibility of a collision. Lane keeping systems (LDW/LKS) increase driving comfort by reducing the risk of unintentionally leaving the lane. This provides greater confidence and peace of mind when driving, especially on motorways. The comfort of night driving is influenced by the intelligent lamp control system (IHC), which automatically adjusts the vehicle’s lighting to the surrounding conditions. The system ensures optimal road illumination without the need to manually switch between high beam and low beam, which is particularly comfortable during long night journeys [38].

When considering the impact of the above-mentioned systems on driving comfort, it can be noted that each of them significantly contributes to reducing driver stress and fatigue. These systems, by automating some driving processes and increasing situational awareness, allow for more relaxing and less stressful driving. However, to maximize their potential, it is necessary to provide drivers with education about the functions and limitations of these systems. It is also worth emphasizing that despite the advancement of these systems, they cannot completely replace the vigilance and skills of the driver, who should remain an active participant in road traffic. 

### 4.3. The Impact of ADASs on the Sense of Safety and Driving Comfort—Survey Study

The research hypothesis set before conducting the study was: “The impact of ADAS on the sense of safety and driving comfort demonstrated by users of these systems”. The study was conducted using a paper survey at the turn of May and September 2023. A total of 80 people (40 women and 40 men) participated in the study. Surveys were distributed to drivers of similar age (between 20–30 years old) and with different experience who use vehicles equipped with ADASs. The survey included questions about the frequency of using individual ADAS functions, their perceived impact on driving safety and comfort, as well as experiences and suggestions regarding these systems.

The survey questionnaire included seven questions, both open and closed. It included the following questions:

1. What type of car do you have?
2. What ADAS systems are installed in your car?
3. How often do you use ADAS systems while driving?
4. Do you think ADAS systems increase your sense of safety while driving?
5. Do ADAS systems affect your driving comfort?
6. Do you have any experiences where ADAS systems helped avoid an accident or dangerous situation?
7. Do you have any comments or suggestions for improving ADAS systems?

One of the first questions in the survey was: What type of car do you have? The choices were: sedan, SUV, hatchback, other. Analysis of the answers to this question allowed me to obtain information about drivers’ preferences regarding the choice of car body type and whether they can be related to the use and perception of ADASs. Respondents who own sedans in my survey constitute a significant percentage of vehicles, as much as 40%. Traditionally associated with comfort and elegance, cars are often equipped with advanced technologies, including ADASs. Sedan owners may be particularly interested in the latest ADASs that improve driving comfort and safety, which is consistent with the general character of these vehicles. SUVs accounted for 35%, known for their higher seating position and spacious interior, and are also often equipped with ADASs. Their users may value both safety and comfort, especially in more difficult road conditions. A total of 15% of respondents had a hatchback. Perhaps this is due to the positioning of these cars as more economical and urban. The remaining 10% were station wagons and coupes. Owners of these vehicles may have specific preferences for ADASs, which may be related to the unique features of their cars, such as larger cargo space or sporty character.

The second question was specific and asked if you had the systems (discussed in the second chapter) in your cars. Adaptive cruise control (ACC)—75% of respondents have it. Its high percentage indicates that it is standard equipment in modern vehicles or an option often chosen by drivers. ACC is particularly appreciated for increasing comfort on long journeys and in congested traffic, reducing the need to constantly change speed. Blind spot detection (BSD), which alerts the driver to vehicles in hard-to-see places, is also widely used. Its presence in 60% of vehicles highlights the importance drivers attach to safety when changing lanes. The lane keeping system (LDW/LKS) is present in 43% of vehicles. This proves that drivers are highly aware of the risks associated with inattention and fatigue. Half of the vehicles are equipped with intelligent lighting control systems, which indicates interest in technologies that improve visibility and safety at night. Steering assistance, often in the form of automatic emergency braking, is found in 40% of vehicles. Although it is not as common as other systems, its presence is important for collision prevention. Analysis of the answers to this question shows that these systems are more and more commonly installed in modern vehicles.

**Figure 7:**
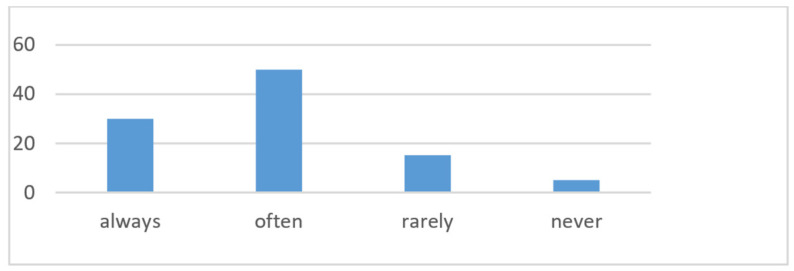
How often respondents use ADASs while driving.

The answers to the third question regarding the frequency of use of ADASs provided information about their acceptance and integration into the everyday driving experience (Figure 7). The “Always” option was selected by 30%—a large group of drivers, which proves a high level of trust and habituation to these technologies. It can be assumed that these users appreciate the benefits of ADAS in terms of safety and comfort. This category may include drivers who often travel long distances or in congested city traffic, where ADASs can significantly contribute to improving the driving experience. Half of the respondents use ADASs “often”, which indicates the widespread acceptance of these technologies. These systems appear to have become an important part of everyday driving for many drivers who are aware of the benefits of ADAS but choose to activate the systems depending on specific road conditions or personal comfort. The “rarely” group of drivers constitutes 15% and may do so for various reasons, such as insufficient awareness of the functions, uncertainty about the effectiveness of the systems, preference for active driving, or specific driving conditions. These users may need additional information or training on the benefits and proper use of ADASs. However, 5% are people resistant to technology and marked “never”. This may be due to a lack of trust in the technology, insufficient knowledge of how it works, or a strong preference for a traditional driving style. The results of the question about the frequency of use of ADASs indicate a generally positive reception of these technologies among drivers. The dominance of the answers “always” and “often” emphasizes that ADASs are considered an important element of support in everyday driving. At the same time, the presence of a group of “rarely” or “never” users highlights the need for further education and improvement of systems to increase their acceptance and effectiveness of use.

**Figure 8:**
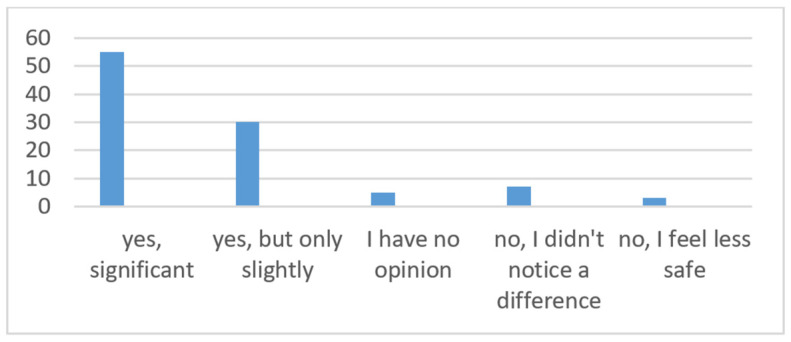
How do respondents feel safe when driving with ADAS systems?

The next question is: Do you think that ADAS systems increase your sense of safety while driving? Answers: Yes, significantly/Yes, but slightly/I have no opinion/No, I don’t notice the difference/No, I feel less safe (Figure 8). Deducing the answer to this question allows you to understand how drivers evaluate these technologies in terms of safety. And “Yes, significantly” was marked by more than half of the respondents, which proves good acceptance and trust in ADASs and the group that believes that these systems significantly contribute to their sense of security. A large number of respondents marked “Yes, but slightly”—30%. These respondents see an improvement in safety thanks to the systems, although they do not consider it significant. This may mean that users are aware of the benefits of ADAS, but their experiences with the systems are not compelling enough to consider the impact to be significant. A total of 5% of respondents are uncertain or have no experience; they cannot determine the impact of ADAS on their sense of security and marked “I have no opinion”. This may be due to lack of experience or sufficient knowledge of these systems. Drivers who choose the answer “No, I don’t notice the difference” are indifferent to technology—7%. They do not notice any difference in the sense of safety when using ADAS. This may suggest that for this group these systems are not effective or understandable enough or that their functionality is not appropriately adapted to their individual needs. A small number of drivers, only 3%, feel less safe with ADASs. This may be related to negative experiences such as false alarms, excessive system intervention, or fear of relying too much on technology. The general analysis of the responses shows that the majority of drivers perceive ADASs as having a positive impact on driving safety, which proves the positive reception of these technologies. At the same time, the presence of a group of users who do not notice the difference or feel less safe highlights the need for further development and optimization of ADASs and user education to maximize benefits and minimize possible negative experiences.

**Figure 9:**
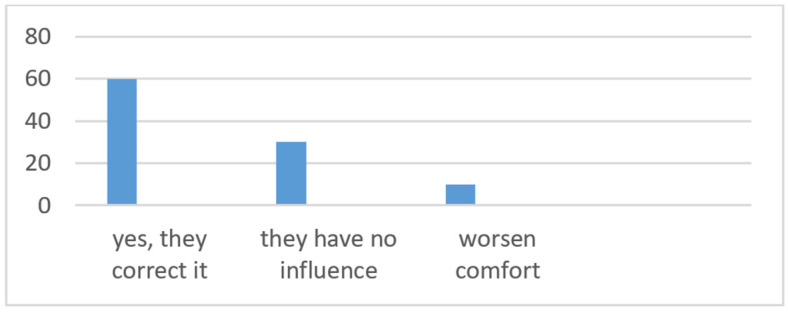
The impact of ADASs on driving comfort according to respondents.

The fifth question on the impact of ADASs on driving comfort reveals the following user observations about these technologies. The answer “Yes, they improve comfort” was indicated by 60% of respondents (Figure 9). Drivers experience increased driving comfort. This may include easier vehicle maneuvering, reduced stress associated with long journeys or congested traffic, and an overall feeling of safety. A high percentage in this category indicates that the systems are perceived as a valuable tool for improving the driving experience. A neutral attitude, i.e., choosing the answer “They have no influence”, was declared by 30% of respondents. This group of drivers does not feel the impact of the systems on driving comfort. This may be due to various factors such as lack of understanding or awareness of ADAS functions, little use of these systems in everyday driving, or personal driving style preferences. These results may suggest the need for greater efforts to educate and promote the benefits of ADASs so that users can fully realize their potential to increase driving comfort. However, a small proportion of respondents believe that driver assistance systems worsen driving comfort. This 10% of negative respondents may be due to various factors, such as false alarms, excessive or inappropriate system intervention, or the feeling that technology is “taking control” from the driver. This result highlights the need to improve systems to make them more intuitive, less invasive, and their operation more understandable and predictable for users. Overall, the responses to the fifth question show that ADASs are perceived by most drivers to improve driving comfort, indicating an overall positive reception of these technologies. However, the presence of a significant number of users who do not perceive an impact on comfort, and a smaller group who believe that these systems impair comfort, highlights the need for continuous development, optimization, and education regarding ADASs. The goal is to maximize benefits for all users and minimize potential negative experiences.

Analysis of the answers to question six regarding direct experiences where ADASs helped avoid an accident or dangerous situation provides information about the practical impact of these technologies on driving safety. Possible answers are yes or no. A large part of respondents, as many as 40%, have positive experiences with the systems and confirm that these systems helped to avoid potential accidents or dangerous situations. This demonstrates the real value of these systems in a practical context, increasing road safety. An increased sense of security can significantly contribute to increasing trust in systems and perceiving them as an essential element of modern vehicles. Users who have experienced direct benefits may be more willing to use these systems and promote their benefits. However, 60% of respondents chose “no” because they had not directly experienced a situation in which the systems would have helped avoid an accident or dangerous situation. This may be due to several reasons, such as limited functionality of the systems in the vehicles, insufficient conditions for activating the systems, or simply the absence of dangerous driving situations. These results highlight the importance of continuous improvement of ADASs and user education to increase their effectiveness and understanding of their capabilities. Ultimately, the goal is clear: to maximize the benefits of ADAS for all road users.

The last survey question concerned comments and suggestions for improving ADASs. It revealed the level of user engagement and satisfaction with these technologies, as well as their willingness to participate in the process of improving them. Possible answers—yes (please describe) or no. The answer “yes” was declared by 35% of respondents, and the most frequently mentioned were: the need for greater precision and reliability of systems, better integration with vehicle functions, and the need for greater personalization and adaptation to the individual preferences of drivers. It revealed specific areas that require improvement, such as increasing the accuracy of the systems, minimizing false alarms, improving the user interface, and better integration with other vehicle systems. The answer “no”, i.e., satisfaction with the systems or no opinion about them, was selected by 65% of drivers. This may suggest general satisfaction with the current state of technology or a lack of sufficient awareness or commitment to make specific proposals for improvement. This result may also indicate that not all users are aware of the potential of ADASs and their limitations, which may result in a lack of specific suggestions for improving them.

### 4.4. Discussion

The survey results confirmed the hypothesis that ADASs are perceived as a tool that improves driving safety and comfort. However, there is a need to further improve these systems to minimize their imperfections, such as false alarms and excessive intervention. Respondents noted that the development of ADAS technology should also go hand in hand with the continuous expansion and modernization of road infrastructure. Road expansion, better and more legible signs, and well-developed information systems will affect the comfort and speed of travel and significantly improve its safety. It is extremely important that systems and infrastructure are constantly improved. Only a systematic and comprehensive approach to road safety will allow for a continuous reduction in the number of road accidents, which will translate into a decrease in risk for all road users. Coordinated action in this area is crucial to ensuring safe and smooth driving on the roads every year.

It is important to remember that ADAS driver-assistance systems have both a direct and indirect impact on driver health, and this impact can be both positive and negative.

1. Positive impacts on driver health:
  - Reduced stress and fatigue: ADAS automates many tasks, such as keeping the vehicle in lane, adaptive cruise control, or automatic emergency braking, which reduces the cognitive load on the driver and reduces the stress associated with driving, especially on long journeys or in city traffic.
  - Increased safety: ADASs help avoid accidents, which directly translates into a reduced risk of physical injury to the driver and passengers.
  - Support in difficult conditions: Systems such as blind spot detection, forward collision warning, or automatic beam adjustment help the driver respond better to changing road conditions, which can reduce stress and the risk of accidents.
2. Potential negative effects:
  - ADAS dependency: Drivers may become overly reliant on assistance systems, which can lead to reduced driving skills and reduced ability to react quickly in emergency situations when ADASs may not function properly.
  - Increased distraction: Automation of certain tasks can lead to drivers being more distracted, such as using phones or other devices, which can increase the risk of accidents in situations where ADASs are unable to respond appropriately.
  - Risk of inaccurate information: Some ADASs may generate false alarms or incorrect warnings, which can lead to unnecessary stress or, in extreme cases, poor driver decisions.
3. Physical health impacts:
  - Exposure to electromagnetic radiation: ADASs use various technologies such as radars, cameras, and sensors that can emit electromagnetic radiation. The health impacts of long-term exposure are not fully understood, although current levels are considered safe.
  - Posture and ergonomic changes: Reduced engagement in driving can lead to a less active driving posture, which can have long-term impacts on physical health, such as spine problems.

ADASs mainly have a positive impact on driver health by improving safety and reducing stress. However, over-reliance on these systems and potential distraction issues can in some cases lead to negative health effects. It is important that drivers are aware of these systems and use them responsibly.

## 5. ADASs Systems Failure

Failure of ADASs can occur in various forms and can have various causes, from technical problems to external conditions. Here are some examples of such failures:

1. Automatic Emergency Braking (AEB) Failure. Description: The AEB system may fail to respond to an obstacle on the road, potentially leading to a collision. This could be due to software glitches, sensor malfunctions, or environmental conditions (e.g., fog, heavy rain) that interfere with sensor performance. Example: In 2020, some car models experienced issues with their AEB systems, leading to false alarms or failure to react in real emergency situations.
2. Lane Keeping Assist (LKA) Failure. Description: The lane keeping assist system may stop working or malfunction, causing unintended lane departures. This could happen due to problems with cameras or sensors failing to detect lane markings properly, particularly in the case of poor lighting conditions, dirty sensors, or poorly maintained road markings. Example: Certain vehicles have been reported to have issues where the system fails to recognize lane markings in rain or bright sunlight, causing the system to deactivate unexpectedly.
3. Adaptive Cruise Control (ACC) Failure. Description: Adaptive cruise control might not correctly adjust the vehicle’s speed in response to traffic. For example, the system may fail to detect a vehicle ahead or may not react to sudden speed changes, increasing the risk of a collision. Example: Some vehicles have experienced problems where the ACC does not respond to sudden stops by vehicles ahead, especially at high speeds on highways.
4. Blind Spot Monitoring (BSM) Failure. Description: The Blind Spot Monitoring system may fail to detect vehicles in the driver’s blind spot, potentially leading to dangerous lane changes. This could be caused by sensor malfunctions, electromagnetic interference, or adverse weather conditions that affect radar performance. Example: There have been cases where the Blind Spot Monitoring system failed to detect vehicles next to the driver’s car, leading to risky maneuvers when changing lanes.
5. Traffic Sign Recognition System Failure. Description: The Traffic Sign Recognition system may fail to correctly identify road signs or provide incorrect information. This could result from software errors, camera issues, or poor weather conditions like rain, fog, or low visibility. Example: In some instances, these systems have incorrectly identified speed limit signs, leading to improper speed suggestions for the driver.
6. False Alarms. Description: ADASs may generate false alarms, warning the driver of non-existent hazards. This can lead to unnecessary stress or risky maneuvers. Example: False collision warnings or false detections of vehicles in the blind spot could cause sudden, unwarranted actions like abrupt braking or lane changes.
7. Software Update Issues. Description: ADASs may malfunction due to errors during software updates. An improperly executed update can cause the systems to operate incorrectly or not at all. Example: After a software update, some ADASs in vehicles might malfunction or fail to activate, requiring service intervention.

These examples illustrate that while ADAS technologies are advanced, they are not immune to failures, which in extreme cases could impact driving safety.

## 6. Comparison of Different ADAS System Architectures

When comparing different architectures with different sensors, the advantages and disadvantages depend on the specific use case, the environment, and the desired outcomes. Let us break down some common architectures and sensors, such as centralized, distributed, and hybrid.

1. Centralized Architecture
  In a centralized architecture, all sensor data are transmitted to a central processing unit for analysis and decision-making. Advantages: Unified data processing: Easier to synchronize and fuse data from multiple sensors; high computational power: The central processing unit can be more powerful, allowing for more complex algorithms; simplified maintenance: Only one core unit needs to be updated or maintained. Disadvantages: Latency issues: High data traffic can lead to communication delays, especially with high-resolution sensors like cameras and LiDAR; single point of failure: If the central processor fails, the entire system can go down; scalability: May not scale efficiently with an increasing number of sensors or sensor types.
2. Distributed Architecture
  In a distributed architecture, sensors have local processing units and only transmit results (or partial data) to a central system. Advantages: Reduced latency: Local processing reduces the need for constant communication with the central unit; scalability: Easier to add more sensors since each sensor handles its own processing; robustness: Failure of one sensor or processing unit does not affect the entire system. Disadvantages: Synchronization challenges: Harder to synchronize data across sensors; increased power consumption: Local processing at each sensor requires more power; Higher cost: each sensor needs to be equipped with local processing capabilities.
3. Hybrid Architecture
  A hybrid architecture combines aspects of centralized and distributed systems, where some sensors have local processing while others rely on the central unit. Advantages: Flexibility: You can choose which sensors need local processing based on the application; optimized performance: Balance between reduced latency and computational power by distributing the workload; fault tolerance: Part of the system can still function if one section fails. Disadvantages: Complexity: More difficult to design and maintain than purely centralized or distributed systems; cost: Can be more expensive to implement, depending on the sensors used.

## 7. Conclusions

In today’s automotive industry, advanced driver-assistance systems play an underestimated role in improving driving safety and comfort. Equipped with complex sensors such as radars, LiDARs, cameras, and ultrasonic sensors, these systems offer drivers unprecedented support by monitoring the vehicle’s surroundings and responding appropriately to detected threats. From adaptive cruise control (ACC) to blind spot detection (BSD), lane keeping assist (LDW/LKS), intelligent lamp control (IHC), and steering assist (EBA), ADAS is the technological foundation of modern vehicles.

A survey conducted among ADAS users showed acceptance and appreciation of the systems as effective tools that increase travel safety and comfort. Most respondents confirmed to me that these systems significantly improve their sense of safety, reduce stress and fatigue, and increase situational awareness on the road. Some drivers shared their direct experiences where ADAS helped avoid potential accidents or dangerous situations, confirming their usefulness. However, this paper also revealed the need for further development and optimization of ADAS. Drivers indicated the need to increase the precision of systems, reduce false alarms, improve the user interface, and better integrate with other vehicle functions. This indicates that while ADASs are a step in the right direction, their evolution must be a continuous process to meet the growing expectations of users. Additionally, there is a significant need to educate drivers about the operation, benefits, and limitations of ADASs. Correctly understanding and applying these systems is crucial to maximizing their potential. This education should cover not only how ADAS works, but also emphasize drivers’ responsibility for driving, even with advanced assistance systems.

The conclusions of the paper emphasize that the future of ADAS depends on the balance between technological progress, personalization of systems to the individual needs of users, and continuous education. As users increasingly trust and rely on ADASs, manufacturers and designers must strive to continually improve them to ensure maximum effectiveness, reliability and user experience. Further development of ADAS has the potential to further increase the safety and quality of travel, making driving not only safer and more enjoyable for all participants, but also vehicle automation. 

## Data Availability

Data are contained within the article.
